# Medicines coverage and community-based health insurance in low-income countries

**DOI:** 10.1186/1478-4505-6-11

**Published:** 2008-10-30

**Authors:** Catherine E Vialle-Valentin, Dennis Ross-Degnan, Joseph Ntaganira, Anita K Wagner

**Affiliations:** 1 WHO Collaborating Center on Pharmaceutical Policy, Harvard Medical School and Harvard Pilgrim Health Care, 133 Brookline Avenue, 6th Floor, Boston, MA 02215, USA; 2 Ecole de Santé Publique, Université Nationale du Rwanda, B.P. 5229 Kigali, Rwanda

## Abstract

**Objectives:**

The 2004 International Conference on Improving Use of Medicines recommended that emerging and expanding health insurances in low-income countries focus on improving access to and use of medicines. In recent years, Community-based Health Insurance (CHI) schemes have multiplied, with mounting evidence of their positive effects on financial protection and resource mobilization for healthcare in poor settings. Using literature review and qualitative interviews, this paper investigates whether and how CHI expands access to medicines in low-income countries.

**Methods:**

We used three complementary data collection approaches: (1) analysis of WHO National Health Accounts (NHA) and available results from the World Health Survey (WHS); (2) review of peer-reviewed articles published since 2002 and documents posted online by national insurance programs and international organizations; (3) structured interviews of CHI managers about key issues related to medicines benefit packages in Lao PDR and Rwanda.

**Results:**

In low-income countries, only two percent of WHS respondents with voluntary insurance belong to the lowest income quintile, suggesting very low CHI penetration among the poor. Yet according to the WHS, medicines are the largest reported component of out-of-pocket payments for healthcare in these countries (median 41.7%) and this proportion is inversely associated with income quintile. Publications have mentioned over a thousand CHI schemes in 19 low-income countries, usually without in-depth description of the type, extent, or adequacy of medicines coverage. Evidence from the literature is scarce about how coverage affects medicines utilization or how schemes use cost-containment tools like co-payments and formularies. On the other hand, interviews found that medicines may represent up to 80% of CHI expenditures.

**Conclusion:**

This paper highlights the paucity of evidence about medicines coverage in CHI. Given the policy commitment to expand CHI in several countries (e.g. Rwanda, Lao PDR) and the potential of CHI to improve medicines access and use, systematic research is needed on medicine benefits and their performance, including the impacts of CHI on access to, affordability, and use of medicines at the household level.

## Background

Low-income countries face considerable challenges in financing health care for their populations: limited tax revenues and complex management of social health insurance translate into enormously inadequate health expenditures per capita with individuals directly absorbing the financial burden of diseases.

Over the past decade, policy makers have presented community financing as an alternative to user fees strategies and a viable option to improving health care systems in low-income countries [1,2]. Several typologies reflect the extreme diversity of community financing, including community cost-sharing, community prepayment, provider based health insurance, and government or social insurance-supported community-driven schemes [3]. The term 'Community-based Health Insurance' (CHI) describes not-for-profit prepayment plans for health care, with community control and voluntary membership that provide risk pooling to low-income populations: these nonprofit (mutual) insurance plans are '*any scheme managed and operated by an organization, other than a government or private for-profit company that provides risk pooling to cover all or part of the costs of health care services' *[4]. The actual CHI contribution to health care financing is difficult to quantify. The ability of CHI to improve financial protection and resource mobilization for health care in poor settings has long been questioned [5,6], but there is now mounting evidence that CHI has positive effects [2-4,7-10]. Objectives, structure, size, and management of CHI plans depend upon local economic and cultural environments. In recent years, CHI plans have multiplied as a transition towards better social protection against the cost of illness in low-income countries where rural populations are usually excluded from other forms of health insurance [11-13]. In countries such as Rwanda [14], Senegal [15], or Lao PDR [16], CHI expansion is an integral component of the national health care financing strategy.

Essential medicines are critical to treating infectious diseases and chronic conditions. Improving access to medicines in developing countries is one key to achieving the Millennium Development Goals [1,17,18]. In high-income countries, health insurers have developed strategies that improve affordability of medicines to their members (purchasing pools, rebates) and increase their appropriate utilization (education of consumers and prescribers) [19]. Conversely, the absence of effective medicines coverage may contribute to the incidence of catastrophic health expenditures [20,21]. Expanding health insurance to improve access to medicines in poor regions of the world is one of the recommendations issued by the 2004 International Conference on Improving Use of Medicines [22]. There, experts from 70 countries recommended expanding health insurance programs to include medicines coverage as a means to improve access to and use of medicines in low-income countries and avoid catastrophic household health care expenditures for the world's poor.

We believe that CHI has the potential to improve access to and quality use of medicines, and that members may be more likely to voluntarily participate in CHI with medicines coverage. To begin to examine this hypothesis, we sought to describe the prevalence of CHI among poor populations; the characteristics of medicine benefits offered by CHI plans; and how medicines coverage may affect the performance of CHI and the satisfaction of members with their plans.

## Methods

To control for the diversity of CHI, we focused our investigation on *low-income *countries as defined by the World Bank, e.g. countries with a Gross National Income of US$875 or less per capita in 2005 [23]. We used data from National Health Accounts (NHA) to evaluate the prevalence of voluntary insurance in health care financing [24]. Because NHA provide only indirect data on CHI, we complemented NHA data with aggregate results from the WHO World Health Survey (WHS), available for 20 of the 54 low-income countries at the time of our study: Bangladesh, Burkina Faso, Chad, Comoros, Côte d'Ivoire, Ethiopia, Ghana, India, Kenya, Lao PDR, Malawi, Mali, Mauritania, Myanmar, Nepal, Pakistan, Senegal, Vietnam, Zambia, and Zimbabwe [25]. Information on out-of-pocket medicine expenditures and voluntary health insurance coverage was extracted from aggregate WHS reports [26].

We performed a literature review looking for data on characteristics of medicines coverage offered by CHI plans in low-income countries. Publications were identified with EMBASE and MEDLINE from January 2002 through December 2006, using the search terms: community-based health insurance, community health planning, cost sharing, developing countries, drug benefit, fees and charges, health expenditures, health insurance, health resources, health services accessibility, insurance, and medicines coverage. We also searched websites of major international organizations such as the International Labor Organization, World Bank, World Health Organization, and websites of international development agencies such as the US Agency of International Development and the *Agence Française de Développement*. Peer-reviewed articles in English and published reports in English or French that identified a CHI plan were selected and classified by country. For each low-income country, we extracted data on the number of schemes, total membership, as well as existence and role of CHI networks. At the individual plan level, we collected information about technical design features related to medicine benefits (i.e., inpatient or outpatient medicines coverage, policies related to use of essential medicines and generics, drug subsidies and donations channeled through plans), organizational incentives related to medicines (i.e., mention of cost-containment tools such as medicine co-payments, strategies for improving quality of prescriptions), or any mention of members' attitudes about how CHI affects access to medicines. When two articles provided the same level of technical information on a scheme, we chose the more recent one.

Finally, to illustrate how individual plans operate, we conducted interviews using a structured questionnaire in two countries where governments have taken the lead in using CHI to expand health insurance coverage to poor populations: one country in Africa (Rwanda) and one in Asia (Lao PDR). This questionnaire is available at .

## Results

### Medicine Expenditures, Poverty, and Voluntary Insurance

Over a third of the world's population resides in low-income countries, where about 70% of people live in rural areas.

According to NHA data (Table 1), out-of-pocket payments account for 85% of private health care expenditures, which themselves represent over half of total health care expenditures in low-income countries. NHA data include information on the existence of prepaid health insurance plans, defined as "*the outlays of private social insurance schemes, commercial and nonprofit (mutual) insurance schemes, health maintenance organizations, and other agents managing prepaid medical and paramedical benefits including the operating costs of these schemes*". Overall, private health care expenditures adjusted for purchasing power parity (ppp) are similar between low-income countries with and without private prepaid plans (ppp-adjusted US $ 36.1 vs. US $ 38.4, respectively). However, in the former, out-of-pocket expenditures constitute a smaller percentage of private health expenditures (78.4% vs. 89.6%), likely due in part to the 5.3% average contribution by private prepaid plans.

**Table 1:** Average per Capita Health Expenditures (HE) in Low-income Countries, WHO National Health Accounts, 2003

**Low income countries**		**Total HE**	**Private HE**	**Prepaid Plans**	**Out-of-Pocket**
			
	n	ppp-adj. US$*	ppp-adj. US$*	% private HE	
**All**	54	68.7	37.5	2.6	85.0
**Countries with private prepaid plans^1^**	22	62.9	36.1	5.3	78.4
**Countries with no private prepaid plans^2^**	32	72.8	38.4	N/A	89.6

* Purchasing power parity adjusted US dollar

^1 ^Bangladesh, Benin, Burkina Faso, Chad, Côte d'Ivoire, Ethiopia, India, Kenya, Lao People's Democratic Republic, Madagascar, Malawi, Mozambique, Niger, Nigeria, Papua New Guinea, Rwanda, Senegal, Togo, Uganda, United Republic of Tanzania, Viet Nam, and Zimbabwe.

^2 ^Afghanistan, Bhutan, Burundi, Cambodia, Central African Republic, Comoros, Democratic People's Republic of Korea, Democratic Republic of the Congo, Eritrea, Gambia, Ghana, Guinea, Guinea-Bissau, Haiti, Kyrgyzstan, Liberia, Mali, Mauritania, Mongolia, Myanmar, Nepal, Pakistan, Sao Tome and Principe, Sierra Leone, Solomon Islands, Somalia, Sudan, Tajikistan, Timor-Leste, Uzbekistan, Yemen, and Zambia.

NHA do not report on categories of population covered by prepaid health insurance plans, but WHS data give some indication about the economic status of privately insured people. WHS data provide information on the overall prevalence of health insurance, and of "voluntary insurance" which includes CHI. Among the 84,135 households interviewed by the WHS in the 17 low-income countries that reported data on the WHS insurance section, a total of 785 respondents reported having some form of health insurance (Table 2). Of those respondents reporting insurance, 61.4% on average were enrolled in voluntary health insurance programs, ranging from 0.8% of insured persons in Pakistan to 90.7% of those insured in Nepal. Coverage by voluntary health insurance was strongly associated with income level: on average, 2% of respondents with voluntary insurance were in the lowest income quintile, while over two-thirds of the insured were in the highest.

**Table 2:** Voluntary Health Insurance in Low-income Countries, World Health Survey, 2003

	**Number of respondents with health insurance**	**Number of respondents with voluntary health insurance**	**Percentage of insured respondents with voluntary health insurance**	**Q1 lowest income**	**Q2**	**Q3**	**Q4**	**Q5 highest income**
		
			Percentage of insured respondents with voluntary health insurance					
		
**Burkina Faso**	40	10	25.0	0.00	0.00	0.00	0.00	100.00
**Chad**	194	17	8.8	0.00	0.00	0.00	10.90	89.10
**Comoros**	272	164	60.3	4.70	8.20	9.90	29.60	47.60
**Cote D'Ivoire**	1244	885	71.1	0.30	0.90	3.80	21.10	74.00
**Ethiopia**	23	4	17.4	0.00	0.00	0.00	0.00	100.00
**Ghana**	359	310	86.4	12.40	11.20	15.10	41.30	20.10
**India**	709	146	20.6	3.10	3.90	6.70	23.30	63.00
**Kenya**	1891	1758	93.0	0.30	3.40	10.20	24.60	61.50
**Malawi**	151	121	80.1	0.00	1.50	5.90	15.70	76.80
**Mali**	172	131	76.2	0.80	17.20	18.80	27.60	35.70
**Mauritania**	208	35	16.8	0.00	0.00	0.00	0.00	100.00
**Nepal**	140	127	90.7	0.00	0.00	0.00	2.00	98.00
**Pakistan**	132	1	0.8	0.00	0.00	0.00	0.00	100.00
**Senegal**	843	320	38.0	0.00	23.70	10.30	26.10	39.90
**Vietnam**	5195	3260	62.8	11.10	16.00	20.60	23.30	29.00
**Zambia**	565	269	47.6	2.70	6.50	14.60	19.70	56.60
**Zimbabwe**	1210	639	52.8	0.70	2.60	3.50	11.60	81.60
		
**Average**	**785**	**482**	**61.4**	**2.12**	**5.59**	**7.02**	**16.28**	**68.99**

**Figure 1:**
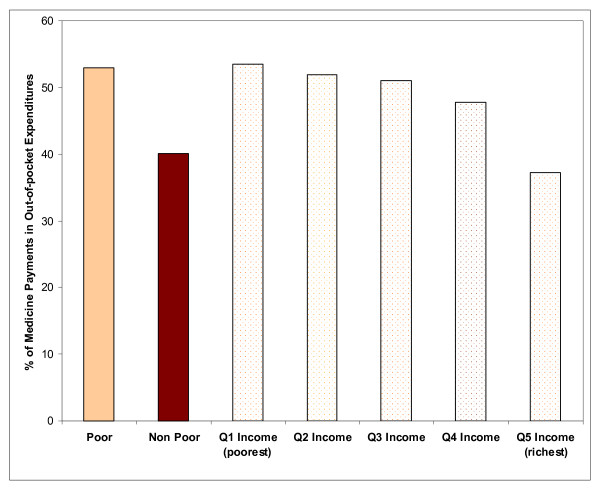
Medicine Payments as a Proportion of Out-of-pocket Expenditures by Income Quintiles in 20 Low-income Countries*, World Health Survey, 2003. * Bangladesh, Burkina Faso, Chad, Comoros, Cote D'Ivoire, Ethiopia, Ghana, India, Kenya, Lao PDR, Malawi, Mali, Mauritania, Myanmar, Nepal, Pakistan, Senegal, Vietnam, Zambia, and Zimbabwe.

WHS data from low-income countries provide some insight about the importance of medicines in household expenditures, and on how economic factors affect access to medicines (Table 3). Medicines constitute the largest reported component of out-of-pocket payments for health care (median: 41.7%; 25%ile, 75%ile: 31.4%, 47.7%), ranging from 11.1% of health expenditures in Chad to 68.8% in Nepal. Furthermore, the proportion of out-of-pocket health expenditures devoted to medicines is inversely associated with income quintiles (Figure 1). Those living below the World Bank poverty standard of US$1 per day devote 53.0% of their health expenditures to medicines, compared to 40.1% among those living above the poverty standard (p < 0.01).

**Table 3:** Distribution of Out-of-pocket Health Payments in Low-income Countries, World Health Survey, 2003

**Out-of-pocket Health Payments**					
	**Medicines**	**Inpatient**	**Outpatient**	**Traditional**	**Others**

	**%**	**%**	**%**	**%**	**%**
					
**Bangladesh**	67.1	6.3	8.1	5.0	13.6
**Burkina Faso**	62.2	9.7	8.0	7.8	12.3
**Chad**	11.1	43.8	12.0	12.2	20.9
**Comoros**	47.6	16.9	15.6	3.0	16.9
**Cote D'Ivoire**	32.2	29.4	15.6	5.5	17.2
**Ethiopia**	43.3	16.8	24.6	2.3	13.0
**Ghana**	40.1	23.3	21.8	5.6	9.1
**India**	44.4	25.4	16.9	3.3	9.9
**Kenya**	31.0	32.8	17.1	1.5	17.5
**Lao PDR**	47.8	25.2	10.1	10.7	6.2
**Malawi**	48.1	15.9	27.1	4.4	4.4
**Mali**	30.8	23.3	20.2	9.9	15.8
**Mauritania**	31.0	22.4	12.1	3.6	30.9
**Myanmar**	47.8	11.9	26.6	5.2	8.4
**Nepal**	68.8	13.9	4.3	1.5	11.5
**Pakistan**	45.5	21.2	14.5	7.0	11.8
**Senegal**	31.5	24.8	13.8	12.9	17.0
**Vietnam**	37.0	27.2	21.9	5.0	8.9
**Zambia**	34.8	26.9	15.2	9.3	13.8
**Zimbabwe**	25.2	12.0	30.6	14.5	17.7

**Median**	**41.7**	**22.9**	**15.6**	**5.4**	**13.3**
25^th ^percentile	31.4	15.4	12.1	3.5	9.7
75^th ^percentile	47.7	25.8	21.8	9.5	17.1

### Paucity of published data about medicines coverage in CHI

We identified fifty-three publications containing information about 1066 CHI plans in 19 low-income countries [10-12,16,27-75]. Table 4 summarizes the number of CHI plans identified and populations covered. Apart from evidence of diversity in size – from seven members in the Khasoko CHI of Kenya [49], to over one million in the Student's Health Home scheme of West Bengal, India [31] – information about individual plans was scarce. Most data were aggregated from either national CHI networks in Lao PDR [16,63] and Rwanda [27,70,71], or from networks affiliated with non-governmental organizations such as La Concertation [73], or Community Health Financing for Eastern Africa (CHeFA-EA) [46,48,49,68]. We found data linking individual schemes to the existence of a medicines benefit package for only 20 schemes: 13 from India, 3 from Bangladesh, 3 from Senegal, and 1 from Benin. Table 5 shows that some form of medicines coverage was documented in 36% of the publications, a medicines co-payment in 32%, outpatient medicine benefits in 25%, an essential medicines list (EML) or generic medicine policies in 21% of the publications. No publication reported the details of medicines benefit packages or how members qualify for coverage; none provided evidence about expanded access to medicines linked with medicines coverage.

**Table 4:** Medicines Coverage and CHI – Literature Review by Country and By Source

**Low-income countries**	**Sources**	**Number of schemes**	**Population covered**	% population covered*
**Rwanda**	*Kalk ^27 ^Kelley ^67 ^PHRPlus Catalogue ^68 ^Jakab ^69^Schmidt ^70 ^Schneider ^71^*	354	3,000,000	33.19
**Mali**	*Diop ^72 ^Drechsler ^12,73 ^Dussault ^74 ^Fischer ^56^Mariko ^57^*	80	198,006	1.46
**Côte d'Ivoire**	*Drechsler ^12,73 ^Dussault, ^74 ^Huber, ^28^*	40	235,280	1.30
**S enegal**	*Atim ^58 ^Bennett ^59 ^Diop ^60,72 ^Drechsler ^12,73^Dussault ^74 ^Jutting ^10^Jakab ^3^*	149	119,300	1.02
**Benin**	*Drechsler ^12,73 ^Dussault ^74 ^Guerin ^61^*	55	33,000	0.39
**Niger**	*Drechsler ^12,73 ^Dussault ^74 ^Meuwissen ^62^*	19	48,700	0.35
**Lao PDR**	*Allianz ^63 ^Ron ^16^*	5	18,829	0.32
**Bangladesh**	*Carrin ^64 ^Letourmy ^65 ^Tabor ^11^*	3	416,209	0.29
**Ghana**	*Baltussen ^66 ^Bennett ^59 ^Diop ^72 ^Sulzbach ^29^*	45	61,600	0.28
**Togo**	*Drechsler ^12,73 ^Dussault ^74^*	25	16,325	0.27
**Guinea**	*Drechsler ^12,73 ^Dussault ^74 ^G autier ^30^*	111	23,844	0.25
**Mauritania**	*Drechsler ^12,73 ^Dussault ^74^*	7	7,635	0.25
**India**	*Devadasan ^31,54–55 ^Jajoo ^32 ^Radermacher ^33^Radermacher ^34 ^Ranson ^35–36,53 ^Sinha ^37^*	13	1,577,544	0.14
**Cambodia**	*Annear ^38^*	5	17,053	0.12
**Burkina Faso**	*De Allegri ^39,51–52 ^Dong ^40–41,46 ^Drechsler ^12,73^Dussault ^74 ^Flessa ^42^*	92	6,100	0.05
**Uganda**	*Dierrennic ^43^, Kiwanuka-Mukiibi ^44 ^Mandelli ^45^PHRPlus Catalogue ^46^Basaza ^75^*	13	8,863	0.03
**Tanzania**	*Kamuzora ^47 ^PHRPlus C atalogue ^48^*	13	8,406	0.02
**Kenya**	*PHRPlus Catalogue ^49^*	30	5,809	0.02
**Chad**	*Drechsler ^12,73 ^Dussault ^74^*	7	930,000	0.01

**Total**		**1,066**	**5,803,433**	

* Using NHA numbers for total population (2003)

**Table 5:** Literature Review – Medicines Coverage and CHI in Low-income Countries

Number of Publications Identifying CHIs in Low-income Countries	53	
	
Number of Publications Describing:		
	
Some Form of Medicine Coverage	19	36%
Medicine Co-Payment	17	32%
Outpatient Medicine Benefit	13	25%
Essential Medicines and Generics Policies	11	21%
Inpatient Medicine Benefit	9	17%
Cost Recovery through Medicine Sales	3	6%
Medicine Subsidies or Donations Funneled Through CBHI	0	
Negotiated Medicine Discounts	0	
Number of Publications Providing:		
	
Detailed Information on Medicine Benefit Packages	0	
Description of Which Members Qualify for Medicines Coverage	0	
Evidence that Coverage Results in Better Access to Medicines	0	

### Two illustrative case studies

In Rwanda, a comprehensive political and legal framework supports the systematic expansion of CHI as the instrument of progression towards universal health insurance coverage [14,76]. Consequently, the number of schemes has rapidly grown in recent years to over 300 in 2006, the highest number of schemes and enrollees in low-income countries (Table 4). In 2005, CHI covered 43% of the entire population, up from 9% in 2003. Local CHI plans and the health centers they support are coordinated by 30 districts. A governmental technical support unit (CTAMS) oversees district units, determines policies and practices of CHI, and dictates common features of benefit packages. Benefits cover low risk events treated at the health center level, including medicines on the national Essential Medicines List (EML), all preventive and curative services, prenatal care, delivery care, laboratory exams, and referral transport to district hospitals. Some selected high-risk events are also covered at the district hospital level. External funding has been used to finance the provision of CHI coverage for the very poor. Interviews with managers of two schemes: *Gicumbi *and *Centre Universitaire de Santé Publique *(*CUSP*) illustrate how CHI functions in Rwanda. These two schemes were selected because they represent two different geographic areas (Northern and Southern Provinces) and two levels of activity (*Gicumbi *coordinates several plans at the district level whereas *CUSP *assumes a strictly local function). Both plans had been in operation for over three years at the time of interviews.

The *CUSP *plan (Huye district, Southern Province) operates at the local level. Created in 2003 in a mixed rural/urban environment, it now insures between 5,000 and 10,000 persons. Membership is voluntary and individual. Yearly premiums are 1,000 Frw (US$1–2) on average. Members pay a fixed co-payment of 100 Frw (US$ 0.20) for every medicine obtained at health centers, and a coinsurance of 10% of retail price for medicines received in the hospital. Medicine costs represented 80% of the total expenditures of *CUSP *in 2006. The *Gicumbi *plan, created in 1999, operates at the district level coordinating 21 plans linked to health centers and 1 plan linked to a hospital, and insuring 85% of the target population. Membership is by family and voluntary, except for the very poor chosen by the community and automatically enrolled without paying premiums. Annual premiums average 3,500 Frw (US$ 6–7). Members pay a fixed 10% coinsurance for all medicines. Outpatient benefits only include medicines on the national EML *and *prescribed by health centers; some medicines not on the EML are covered by the inpatient benefit. At the district level, the *Gicumbi *district/regional authority buys medicines from the Rwanda central pharmacy (CAMERWA) at average wholesale price plus 5% margin and sets retail prices. Medicines expenditures of the *Gicumbi *plan increased by a factor of five after implementation of the Ministry of Health decision to cover the very poor, suggesting a broader access to both care and medicines by these populations. Most frequently used medicines are amoxycillin, paracetamol, quinine, cotrimozaxole, and penicillin V.

According to the interviews, *Gicumbi's *policy of familial membership and free-of-charge enrollment of some of the poorest in the community indicates an emphasis on equity in access of care. Its outpatient drug coverage restrictions suggest an effort to control the quantity and appropriateness of medicines used that may be beneficial to its long-term financial sustainability. On the other hand, *CUSP's *policy of individual membership for everyone without subsidy may be less oriented towards equity. Its strategy of fixed co-payment for every medicine obtained at the health center without coverage restrictions may target over utilization without trying to influence the choice of medicines.

Like Rwanda authorities, those of Lao PDR have adopted a national strategy to increase health resource mobilization for the poor. In April 2005, regulations for a nationwide implementation of community based health insurance were issued after three pilot CHI projects showed promising results [16]. However, interviews confirmed that the current expansion is still at a very early stage. By late 2006, a total of 3,368 families were enrolled in five CHI schemes. Regulations seem to leave little autonomy to communities in day-to-day operations. Regardless of schemes, CHI membership is voluntary. Family premiums are set by the government (average US$ 2.5–3.0 per family per month), and differ between urban and rural areas. Members receive medicines free of charge as long as these are on the national EML. District hospitals, which are the primary point of care, receive monthly capitation payments from CHI plans for enrollees, and have to report every month to a district management committee on utilization and drug consumption. In one scheme, medicine costs in 2006 amounted to five million kip/month (about US$ 500), representing 62.5% of its total expenditures. Plans have not extended coverage to the very poor yet. One interviewed official noted a high demand for care among CHI members, who tend to expect premiums to be paid back in form of services. The voluntary nature of membership was identified as a challenge, because it leads members to leave schemes when they do not have enough money to pay premiums, are dissatisfied with the quality of services provided, or perceive no need for care.

## Discussion

We combined three complementary sources of data to explore CHI medicines coverage in low-income countries. Our findings highlight the paucity of evidence about medicines coverage and medicines utilization in community-based health insurance programs, as well as the need for understanding better the role of medicines coverage in the overall health care financing strategy of low-income countries. Several factors make systematic analysis of data about the structure and processes of CHI plans particularly challenging: small size, diversity of communities where they function, and lack of infrastructure or technical capacity. Indeed, most available data come from schemes currently receiving assistance from governments, large micro-insurance networks, or international organizations[71,77] A plausible explanation could be that the focus to date has been on strategic expansion and political acceptance of CHI rather than on CHI performance. The scaling up of CHI plans is gaining momentum in several countries, and more systematic data may become available. It will then be possible to collect and analyze data on the availability and performance of medicines benefits with the objective of improving the effectiveness of CHI plans.

The paucity of data may also reflect the fact that many CHI plans operate in environments where drug supply systems are so weak that covering medicines is not a realistic option. Nevertheless, as WHS data demonstrate, payments for medicines constitute about half of out-of-pocket expenditures in low-income countries, and this proportion increases with poverty. Even in settings where medicines supply may not be adequate, poor people still use a substantial portion of their meager resources to buy medicines.

Overall, our findings confirm that voluntary insurance, which includes CHI, has a very low penetration in low-income countries where private payments, representing over half of health care expenditures, are mostly made of out-of-pocket. Our literature review identified CHI plans in only one-third of low-income countries (19/54); in only four countries did the plans cover more than 1% of the population. Reasons may vary with national contexts. For instance in Vietnam, the recent emphasis by the Social Security Agency on extending social health insurance coverage to poor populations is consistent with the relatively high percentage of voluntarily insured Vietnamese in the lowest income bracket (11.1%), and may explain why CHI has not been the focus of attention in this country in recent years [78-81]. WHS data in several countries as well as interviews with CHI officials in Lao PDR and Rwanda show that CHI plans experience substantial difficulties in reaching poor people: only 2% of WHS respondents enrolled in voluntary insurance belong to the lowest income bracket. This, and the fact that we were unable to identify CHI schemes in two-third of low income countries, indicate that at present CHI reaches a minute proportion of the underprivileged [12,73].

The raison d'être of CHI is to improve access to care for the poorest of the society. With low penetration among the poor, CHI does not yet achieve this goal. We believe that a focus on inpatient and outpatient medicines coverage, adjusted to local circumstances, may be one strategy to encourage higher rates of voluntary enrollment in CHI by the poor. CHI plans which cover medicines can devise strategies to ensure that quality medicines are consistently available at health centers, provide incentives (such as lower copayments) to use generics, and encourage appropriate prescribing through education and administrative systems. A key challenge will be to ensure financial sustainability of the CHI plans as enrollment and demand for medicines increase.

To be successful, CHI must perform well in two areas: revenue collection and strategic purchasing [8]. Medicines coverage policies have an important influence on both. Low voluntary enrollment is a major obstacle to scaling up CHI. Studies have shown that perceptions about quality of care affect CHI membership: coverage for and availability of medicines may be a key determinant of these perceptions [57,77,82]. For example, some schemes explicitly exclude expensive antiretroviral or tuberculosis medicines from their benefits [11], or sell low-cost drugs at up to seven times the international retail price to recover other costs [27]. Such coverage and pricing policies may deter enrollment, and work against effective performance. CHI plans can also boost direct revenues by channeling subsidies from public authorities and international donors, which seems to be the case in Rwanda. This approach has the potential to increase the ratio of prepaid contributions to health care costs, an indicator of financial protection; and to improve the financial stability of plans, allowing them to play a more active role in supporting premiums of the poorest, thereby reaching greater equity in access to medicines, another key performance domain for CHI [8].

Financial access to medicines can be expanded by integrating evidence about effectiveness, preferences of providers, and medical needs of members in decisions about which medicines to cover, negotiating affordable prices with providers, and working with local medicines outlets to ensure availability of medicines. The potential of CHI to influence prescription behavior and to control medicine costs is real. Even if they do not purchase medicines directly, CHI plans can negotiate payments with those who purchase and supply medicines, and they can design incentives to use recommended medicines. In Benin, the *Association d'Entraide des Femmes *scheme preferentially contracts with religious health care providers, because these providers receive donations of brand name medicines that patients prefer, and sell them at discounted prices [61]. While this policy may be rational in achieving reliable supply and low prices in the short-term, the commitment to brand name rather than generic medicines and reliance on donations may be uneconomical and unsustainable as a long-term strategy.

From our review, it is clear that appropriate tools to assist in designing and managing medicines benefit packages adapted to low-income environments do not exist yet, despite the multiplicity of workshops and manuals targeting CHI administrators [83,84]. Training CHI managers to analyze dispensing data in order to better understand and manage medicines utilization, as is done in high-income countries, should be a high priority. CHI managers can play a critical role if they are aware of medical needs of their members, are familiar with local care seeking and prescribing patterns, and understand local drug pricing and supply management issues. For example, incentives to prescribe and use generics may help to shift market share away from commonly used brand medicines, exert pressure to reduce the prices of these brand products, and increase treatment affordability for both CHI plans and patients.

The paucity of medicines coverage data prevented us from examining the role of CHI on improving access to medicines. Yet, interviews suggest that medicines represent between 65% and 80% of costs incurred by some plans, highlighting the importance of monitoring medicines utilization and expenditures in CHI. Medicines coverage policies can be used to control medicine expenditures and to improve quality use by rewarding adequate prescription behavior and by encouraging the use of effective and safe medicines at lowest possible prices. In poor communities, CHI provides a critical institutional link between patients, providers, and suppliers of medicines: it can play a key role by negotiating with medicine suppliers, by adjusting medicines coverage to local health care priorities, by disseminating education materials tailored to the community about quality use of medicines, by linking medicines coverage to treatment adherence, and by rewarding providers and community-based workers who follow treatment guidelines. In this context, the large-scale social experiment of CHI development in Rwanda is of significance, since it may bring important progress to the understanding of challenges faced by CHI in low-income settings. Interviews with CHI administrators in Rwanda illustrate the interplay of government support and community involvement needed to design medicine benefits for the poor. Much more could be learned in this setting by comparing membership and utilization among plans with different medicine benefits, and by exploring the relationship between medicines coverage and enrollment, cost recovery, and financial stability.

## Conclusion

In conclusion, our results show that the extent and nature of medicines coverage offered by CHI in low-income countries are not well-reported at this time. Lack of access to medicines is a crucial issue in the developing world. We believe that better medicines coverage and well-designed medicine benefits are part of a strategy to address this problem. Further research is needed to characterize current coverage and utilization of essential medicines in CHI, to identify effective medicines benefit packages in low-income settings, and to study the impacts of these benefits on patterns of service utilization and clinical outcomes. A better understanding of medicine policies in CHI can help national policymakers and insurers who develop strategies to improve health care financing systems, prevent catastrophic health expenditures, and achieve the Millennium Development Goals. To that effect, the Rwanda experience may constitute a unique opportunity to evaluate the contribution of CHI in improving access of medicines, medicines utilization, and ultimately health outcomes in low-income environments.

## Competing interests

The authors declare that they have no competing interests.

## Authors' contributions

CVV carried out the literature review, designed the structured interviews, and drafted the manuscript. DRD conceived of the study, participated in its design and reviewed the manuscript. JN provided input during the initial phase of the project, carried out interviews in Rwanda, and reviewed the manuscript. AW participated in the design of the study, carried out an interview with three officials from the National Institute of Public Health, the Ministry of Health, and the WHO office in Lao PDR, and reviewed the manuscript.
